# Protective Role of Natural and Semi-Synthetic Tocopherols on TNFα-Induced ROS Production and ICAM-1 and Cl-2 Expression in HT29 Intestinal Epithelial Cells

**DOI:** 10.3390/antiox10020160

**Published:** 2021-01-22

**Authors:** Vladana Domazetovic, Irene Falsetti, Caterina Viglianisi, Kristian Vasa, Cinzia Aurilia, Maria Stio, Stefano Menichetti, Teresa Iantomasi

**Affiliations:** 1 Department of Biomedical, Experimental and Clinical Sciences “Mario Serio”, University of Florence, Viale Pieraccini, 6, 50134 Firenze, Italy; vladana.domazetovic@unifi.it (V.D.); irene.falsetti@unifi.it (I.F.); aurilia.cinzia@gmail.com (C.A.); maria.stio@unifi.it (M.S.); 2 Department of Chemistry “Ugo Schiff”, University of Florence, Via Della Lastruccia, 3-13, 50019 Sesto Fiorentino (FI), Italy; caterina.viglianisi@unifi.it (C.V.); kristian.vasa@unifi.it (K.V.)

**Keywords:** oxidative stress, natural and semi-synthetic tocopherols, tumor necrosis factor-α (TNFα), intercellular adhesion molecule-1 (ICAM-1), claudin-2 (Cl-2)

## Abstract

Vitamin E, a fat-soluble compound, possesses both antioxidant and non-antioxidant properties. In this study we evaluated, in intestinal HT29 cells, the role of natural tocopherols, **α-Toc** and **δ-Toc**, and two semi-synthetic derivatives, namely *bis*-δ-Toc sulfide **(δ-Toc)_2_S** and *bis*-δ-Toc disulfide **(δ-Toc)_2_S_2_**, on TNFα-induced oxidative stress, and intercellular adhesion molecule-1 (ICAM-1) and claudin-2 (Cl-2) expression. The role of tocopherols was compared to that of *N*-acetylcysteine (NAC), an antioxidant precursor of glutathione synthesis. The results show that all tocopherol containing derivatives used, prevented TNFα-induced oxidative stress and the increase of ICAM-1 and Cl-2 expression, and that **(δ-Toc)_2_S** and **(δ-Toc)_2_S**_2_ are more effective than **δ-Toc** and **α-Toc**. The beneficial effects demonstrated were due to tocopherol antioxidant properties, but suppression of TNFα-induced Cl-2 expression seems not only to be related with antioxidant ability. Indeed, while ICAM-1 expression is strongly related to the intracellular redox state, Cl-2 expression is TNFα-up-regulated by both redox and non-redox dependent mechanisms. Since ICAM-1 and Cl-2 increase intestinal bowel diseases, and cause excessive recruitment of immune cells and alteration of the intestinal barrier, natural and, above all, semi-synthetic tocopherols may have a potential role as a therapeutic support against intestinal chronic inflammation, in which TNFα represents an important proinflammatory mediator.

## 1. Introduction

**Figure 1:**
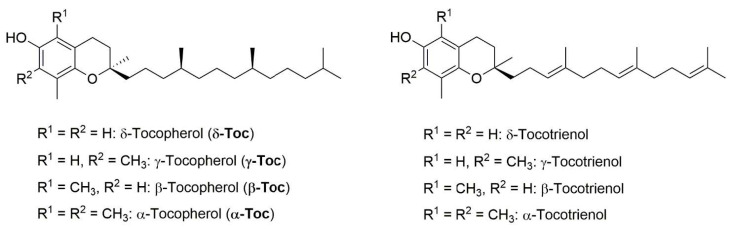
Structure of α-, β-, γ-, and δ-tocopherol (**α-**, **β-**, **γ-,** and **δ-Toc**), and the corresponding α-, β-, γ-, and δ-tocotrienol.

Vitamin E, the most important fat-soluble vitamin, is a mixture of four tocopherols **(Toc) (α-, β-, γ-, and δ-Toc)** and four tocotrienols (α-, β-, γ-, and δ-tocotrienol), differing in the number and position of methyl groups on the chromanol ring [1], and the saturation of the aliphatic tail (Figure 1). As a matter of fact, α-tocopherol **(α-Toc)** is the main component of natural vitamin E, and is often indicated as vitamin E itself.

Dietary sources of vitamin E are fat-containing foods in which different tocopherols and tocotrienols are present in variable quantity [2]. However, daily foods, such as cheese, fruits, and eggs, also contain isoforms of vitamin E [3]. Vitamin E is considered a powerful antioxidant for its ability to scavenge radical oxygen species (ROS), and, due to its membrane localization, protects cell membranes from lipid peroxidation [4]. In fact, membrane phospholipids are targets easily exposed to oxidants, and the absence of vitamin E does not promote damage repair [2]. Oxidative stress is a key event involved in the onset and progression of the inflammatory response that characterizes pathologies such as cardiovascular diseases, type 2 diabetes mellitus, atherosclerosis, autoimmune diseases, and intestinal inflammatory disorders [5,6,7,8,9]. In fact, pro-inflammatory mediators, including tumor necrosis factor-α (TNFα), increment intracellular production of ROS, thus causing cellular damage. This also occurs in epithelial intestinal cells in response to the oxidative stress associated with chronic intestinal bowel disease (IBD), enteritis, and colon cancer [10]. Oxidative stress and inflammatory mediators alter intestinal barrier function and disrupt tight junctions by inducing changes in localization and expression of tight junctional proteins, such as occludins, cadherins, and claudins (Cls), among which are Cl-1, Cl-2, and Cl-7 [11,12,13]. Moreover, in the intestine they are also involved in the increase of adhesion molecules, such as intercellular adhesion molecule-1 (ICAM-1), that contribute to the enhancement of trafficking leukocytes to inflammatory sites [14,15,16]. In particular, TNFα, the cytokine with a key role in the regulation of inflammatory processes in IBD [17], induced ROS increase, as well as ICAM-1 and Cl-2 expression up-regulation, in human epithelial colorectal adenocarcinoma cell line, HT29 cells [18,19]. Vitamin E protects the intestinal barrier against oxidative stress damage [20,21], and it has an important role in the prevention of various pathologies, due to its antioxidant and anti-inflammatory action, and in down-regulating cell adhesion molecules, inhibiting platelet aggregation, and increasing nitric oxide synthase activity [22,23,24]. Indeed, vitamin E is able to promote positive biological responses independently of its antioxidant properties; being involved in the redox-independent regulation of gene expression, inhibition of protein kinase C expression and activity, as well as cell proliferation [1]. Overall, to vitamin E has been attributed anti-cancer properties [25], and the ability to stimulate cellular immune response [26] and reduce the progression of Alzheimer’s disease in patients with moderate pathology [27]. In fact, in conditions of vitamin E deficiency, anemia, neuromuscular and cardiovascular problems, and defective immune response can occur, causing heart disease, myopathies, and nerve damages [2]. Vitamin E deficiency is often present in IBD patients, and this can be due to bowel ulcerations and/or resection, which decrease the intestinal absorptive surface [28]. Administration of vitamin E reduces the development of experimental colitis in animal models [29,30], and semi-synthetic derivatives of vitamin E can also act as anti-inflammatory agents, inhibitors of nuclear factor-kappa B (NF-kB) translocation, and adjuvants in chemotherapeutic treatments [31,32,33].

**Figure 2:**
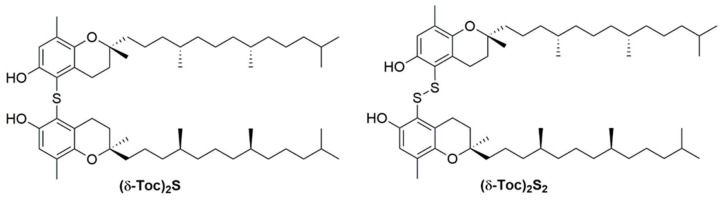
Structure of *bis*-δ-tocopheryl sulfide **(δ-Toc)_2_S** and *bis*-δ-tocopheryl disulfide **(δ-Toc)_2_S_2_** tested in this study.

The aim of this study was to investigate the protective role of natural, **α-Toc** and **δ-Toc**, and semi-synthetic, *bis*-δ-tocopheryl sulfide and *bis*-δ-tocopheryl disulfide, indicated as **(δ-Toc)_2_S** and **(δ-Toc)_2_S_2_**, respectively [34], (Figure 2), on TNFα-induced oxidative stress, and ICAM-1 and Cl-2 expression, in HT29 cells.

Additionally, the relationship between the intracellular redox state and the expression of ICAM-1 and Cl-2 was evaluated in the same cells. To clarify the actual role of the antioxidant activity of the derivatives tested, the same experiments were performed in the presence of *N*-acetylcysteine (NAC), an antioxidant precursor of glutathione, known to be able to reduce intracellular oxidative stress [35].

## 2. Materials and Methods

### 2.1. Cell Culture and Treatment

HT29 cells, obtained from the American Type Culture Collection (Manassas, VA, USA), were grown at 37 °C in a 5% CO_2_ atmosphere in McCoy’s 5A modified medium supplemented with 10% fetal bovine serum, 72 mg/L penicillin and 100 mg/mL streptomycin. Experiments were performed in cells seeded in 12-well plates, and at 80–90% confluence pre-treated or not for 1 h with various concentrations (5–100 µN) of natural, **α-Toc** and **δ-Toc**, and semi-synthetic, **(δ-Toc)_2_S** and **(δ-Toc)_2_S_2_**, or for 16 h with 20 mM NAC. Worthy of mention is the use of normal concentration (µN) for the tocopherol derivatives. Since **(δ-Toc)_2_S** and **(δ-Toc)_2_S_2_** contain two δ-tocopherol units, we decided to compare the effect of α-Toc and δ-Toc with the same concentration of tocopherol units in the sulfide and disulfide under investigation. In other words, the molar concentration of **(δ-Toc)_2_S** and **(δ-Toc)_2_S_2_** used in these experiments was ½ of that of **α-Toc** and **δ-Toc**. Subsequently, these cells were stimulated or not for another 24 h with 10 ng/mL TNFα. The vitamin E isoform concentration range included those reported in the literature [36,37]. We utilized concentrations as low as 5 and 10 µN, and as high as 50 and 100 µN. Then, 0.7 *N* solutions of **α-Toc**, **δ-Toc**, **(δ-Toc)_2_S**, and **(δ-Toc)_2_S_2_** in ethanol were diluted with phosphate bufferedsaline (PBS) in order to reach the required concentration of antioxidant to be added to the cells. A final homogenous solution was achieved in all cases, and 0.008% ethanol was added to the respective vitamin E-untreated cells. All reagents used for cell culture and stimulation were purchased from Sigma-Aldrich (St. Louis, MO, USA).

### 2.2. Cell Viability by Trypan Blue Dye Exclusion Test

Cell viability was measured in HT29, treated or not with the highest concentrations (50 and 100 µN) of natural, **α-Toc** and **δ-Toc**, and semi-synthetic, **(δ-Toc)_2_S** and **(δ-Toc)_2_S_2_**, or 20 mM NAC, as above described, and stimulated for 24 h with 10 ng/mL TNFα. Cells, detached with trypsin and collected by centrifugation at 130× *g* for 10 min, were resuspended in cold phosphate buffer saline (PBS). Next, 40 µL cell suspension was mixed with equal parts of 0.4% trypan blue dye, loaded into a Neubauer chamber, and viewed under a microscope. The number of dead (stained) and live cells (unstained) was manually counted. The percentage of viable cells was calculated considering the ratio between number of unstained cells and number of total cells ratio ×100.

### 2.3. Bis-δ-Tocopheryl Sulfide **(δ-Toc)_2_S** and Bis-δ-Tocopheryl Disulfide **(δ-Toc)_2_S_2_** Synthesis

*Bis*-δ-tocopheryl sulfide **(δ-Toc)_2_S** and *bis*-δ-tocopheryl disulfide **(δ-Toc)_2_S_2_** were prepared by regioselective sulfenylation of **δ-Toc** with the phthalimidesulfenyl chloride PhtNSCl (Pht = Phthaloyl). The *ortho*-hydroxy-*N*-thiophthalimide derivative obtained was used as common starting material for the synthesis of both compounds. *Bis*-δ-tocopheryl sulfide **(δ-Toc)_2_S** was the product of the reaction of *ortho*-hydroxy-*N*-thiophthalimide and δ-Toc in the presence of triethylamine [34], and *Bis*-δ-tocopheryl disulfide **(δ-Toc)_2_S_2_** was the result of the reaction of the *ortho*-hydroxy-*N*-thiophthalimide with *bis*-trimetylsilyl selenide (Me_3_Si)_2_Se [38]. Both compounds were carefully purified by silica gel flesh column chromatography, and fully characterized [34]. 

### 2.4. Intracellular ROS Production Assay

The intracellular production of ROS was detected, as previously described [39], by using 2′-7′-dichlorodihydrofluorescein diacetate (H2DCF-DA) (Invitrogen, Carlsbad, CA, USA), a fluorogenic dye, which within the cells is deacetylated by esterase to a non-fluorescent compound, subsequently oxidized by ROS into the fluorescent 2′-7′-dichlorodihydrofluorescein (DCF). A total of 5 mg/L (H2DCF-DA) was added to HT29 cells, treated as described above, 30 min before the end of TNFα stimulation. HT29 cells were washed and lysed in a buffer containing 50 mM Tris/HCl pH 7.5, 1% Triton X-100, 150 mM NaCl, 100 mM NaF, and 2 mM EGTA. Fluorescence intensity was measured using a Fluoroskan AscentFL microplate reader (Thermo Fisher Scientific, Waltham, MA, USA) at 485 nm excitation and 518 nm emission wavelengths. Data, normalized on total protein content, were expressed as the percentage of ROS detected in untreated and unstimulated cells (control). 

### 2.5. ICAM-1 and Cl-2 Assay

ICAM-1 and Cl-2 expression was detected by measuring intracellular ICAM-1 and Cl-2 levels in the cell lysates of HT29 cells, treated as described above, using a Human ICAM-1 ELISA kit (Uscn Life Sciences Inc., Wuhan, Hubei, PRC) and Human Claudin-2 ELISA kit (MyBiosource, San Diego, CA, USA), in accordance with the manufacturer’s instructions. Cell lysates were obtained by HT29 detached with trypsin, and collected by centrifugation at 130× *g* for 10 min. Cells, washed three times and resuspended in cold PBS, were ultrasonicated four times for 15 s. Subsequently, the cell lysates were centrifuged at 1500× *g* for 10 min to remove cellular debris, and the supernatants were used to perform ICAM-1 and Cl-2 assays. Data, normalized on total protein content, were expressed as percent of ICAM-1 or Cl-2 levels detected in controls. 

### 2.6. Protein Assay

Protein concentration was detected by the bicinchoninic acid solution (BCA) protein reagent assay (Thermo Scientific, Waltham, MA, USA), using bovine serum albumin (Sigma-Aldrich) as a standard [40].

### 2.7. Statistical Analysis

GraphPad Prism software was used to determine the statistical significance of the differences by one-way ANOVA analysis with Bonferroni’s multiple comparison test. All experiments were carried out three times, and data are expressed as the mean ± SEM. *p* ≤ 0.05 was considered statistically significant.

## 3. Results

### 3.1. Effect of Natural **α-Toc** and **δ-Toc**, Semi-Synthetic, **(δ-Toc)_2_S** and **(δ-Toc)_2_S_2_**, and NAC on HT29 Cell Viability

**Figure 3:**
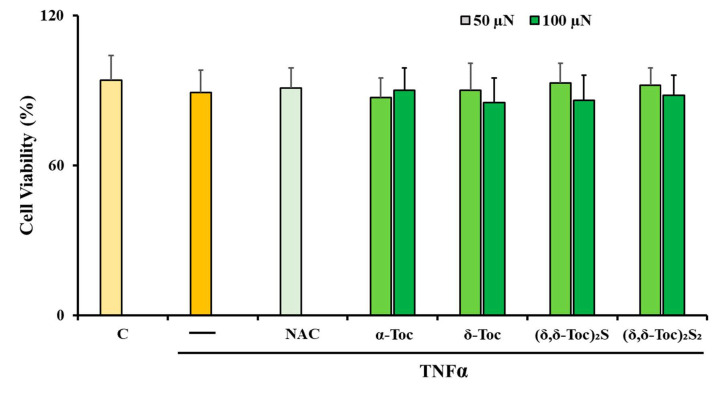
Viability of HT29 pre-treated with natural, **α-Toc** and **δ-Toc**, semi-synthetic, **(δ-Toc)_2_S** and **(δ-Toc)_2_S_2_**, or NAC, and stimulated with TNFα. HT29 cells, pre-treated or not with 50 and 100 µN of **α-Toc**, **δ-Toc**, **(δ-Toc)_2_S**, **(δ-Toc)_2_S_2_**, or 20 mM NAC, as reported in Materials and Methods, were stimulated or not for 24 h with 10 ng/mL TNFα. Cell viability was performed by Trypan blue dye exclusion test. Percentage of viable cells was calculated considering the ratio between the number of unstained cells and the number of total cells ×100. Values are the mean ± SEM of three experiments repeated in triplicate.

Figure 3 shows that cell viability did not undergo significant changes in HT29 stimulated with TNFα, both in the absence and in the presence of NAC, or the highest concentrations used (50 and 100 µN) of natural, **α-Toc** and **δ-Toc**, and semi-synthetic, **(δ-Toc)_2_S** and **(δ-Toc)_2_S_2_**. This indicates that, in our experimental conditions, cell viability was not altered, and the various tocopherol derivatives were not cytotoxic. In this light, in further experiments, all tocopherols were used at these or lower concentrations.

### 3.2. Effect of Natural **α-Toc** and **δ-Toc**, Semi-Synthetic, **(δ-Toc)_2_S** and **(δ-Toc)_2_S_2_**, and NAC on TNFα-Induced ROS Production in HT29 Cells

**Figure 4:**
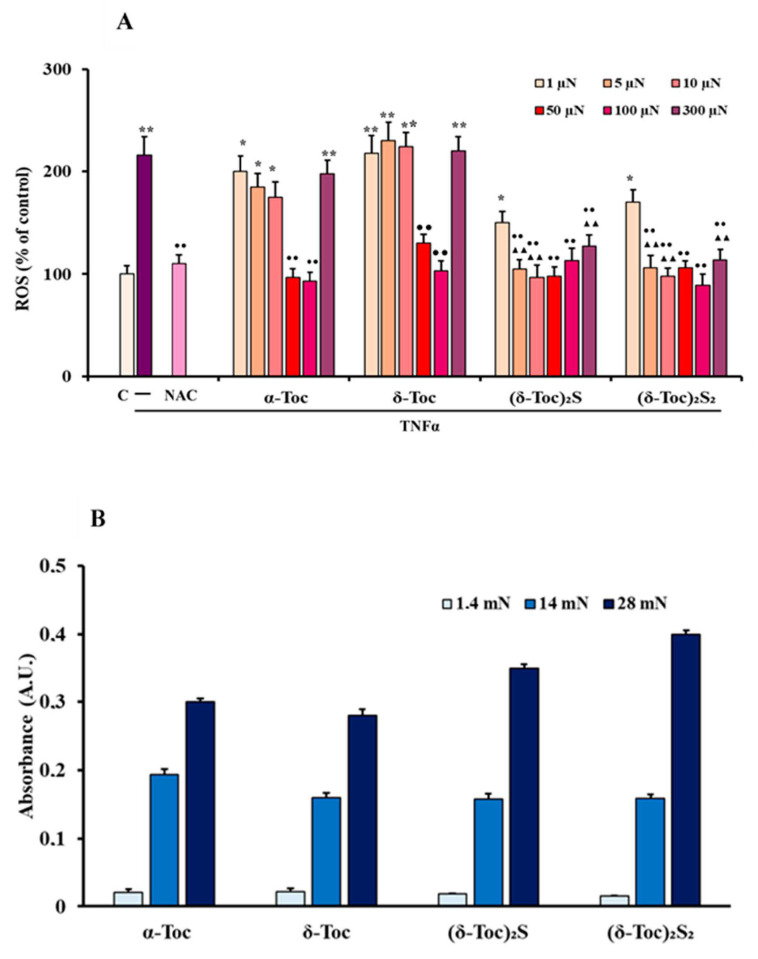
Intracellular ROS production in HT29 cells pre-treated or not with natural, **α-Toc**, **δ-Toc**, semi-synthetic, **(δ-Toc)_2_S**, **(δ-Toc)_2_S_2_**, or NAC, and stimulated with TNFα (**A**) and solubility of tocopherols (**B**). HT29, pre-treated or not with various concentrations (1–300 µN) of **α-Toc**, **δ-Toc**, **(δ-Toc)_2_S**, **(δ-Toc)_2_S_2,_** or 20 mM NAC, as reported in Materials and Methods, were stimulated or not for 24 h with 10 ng/mL TNFα. The intracellular ROS production was assayed by measuring the fluorescence intensity of the intracellular oxidation-sensitive probe H2DCFDA. The values, normalized on total protein content and expressed as the percentage of untreated and unstimulated cells (control, C), are the mean ± SEM of three experiments repeated in triplicate. Solubility of natural and semi-synthetic tocopherols was detected at concentrations (1.4–28 mN) obtained by diluting in PBS 0.7 N solutions in ethanol. Relative absorbance was measured at 292 nm. Data are the mean ± SEM of three experiments, and are expressed as arbitrary unit (A.U). * *p* ≤0.05; ** *p* ≤ 0.001 compared to C cells; ^●●^ *p* ≤ 0.001 compared to untreated and TNFα-stimulated cells; ^▲^^▲^ *p* ≤ 0.001 compared to **α-Toc** and **δ-Toc** pre-treated, and TNFα-stimulated cells.

Oxidative stress was detected by measuring intracellular ROS production in TNFα-stimulated HT29 cells pre-treated or not with various concentrations (5–100 µN) of **α-Toc, δ-Toc, (δ-Toc)_2_S, (δ-Toc)_2_S_2_**, or 20 mM NAC. Figure 4 shows that TNFα increased significantly intracellular ROS levels, by about 120%, as compared to control cells, and that this ROS increase did not occur in NAC pre-treated HT29 cells. Tocopherols pre-treatment did not prevent TNFα-induced ROS production in a concentration-dependent manner. In fact, pre-treatment with low concentrations of **α-Toc** and **δ-Toc** (5 and 10 µN) did not prevent a TNFα-induced ROS increase in TNFα-stimulated HT29 cells. Conversely, when **α-Toc** and **δ-Toc** were used at high concentrations (50 and 100 µN) they totally prevented ROS production (Figure 4). **(δ-Toc)_2_S** and **(δ-Toc)_2_S_2_** were able to inhibit the increase of ROS levels in TNFα-stimulated cells, both at low and high concentrations (5–100 µN) (Figure 4). Moreover, it is notable that **(δ-Toc)_2_S** and **(δ-Toc)_2_S_2_** at all concentrations used, as well as **α-Toc** and **δ-Toc** at 50 and 100 µN, prevented TNFα-induced ROS production similarly to NAC. To better clarify the trend of the dose–response curve, we detected ROS values in HT29 pre-treated with a lower and a higher concentration of tocopherols. Figure 4 shows that the pre-treatment of HT29 cells with 1 µN or 300 µN **α-Toc** and **δ-Toc** did not prevent TNFα-induced ROS increase. While data obtained with 1 µN concentration are perfectly in line with previously discussed data, the increase of ROS using 300 µN concentration of natural **α-Toc** and **δ-Toc** seems in accordance with the already reported and intriguing pro-oxidant role of vitamin E observed in several experiments, particularly when used at high concentrations [41,42,43,44].

On the other hand, semisynthetic **(δ-Toc)_2_S** and **(δ-Toc)_2_S_2_** showed a limited decrease of ROS values in TNFα-stimulated HT29 cells, even when used at 1 µN concentration. Moreover, when they were used at 300 µN the ROS values were similar to those measured in the range 5–100 μN, and with a limited increase if compared with those observed for **α-Toc** and **δ-Toc**. These data, confirming the beneficial effect exerted by our semi-synthetic derivatives in a huge range of concentrations, support the lack of a direct dose–effect relationship, either for **(δ-Toc)_2_S**, **(δ-Toc)_2_S_2_** in all concentration ranges tested, or for **α-Toc** and **δ-Toc** in the 5–100 μN concentration range. As described in Section 2.1, to reach the concentrations used of semi-synthetic and natural tocopherols in the cells, their 0.7 N solutions in ethanol were subsequently diluted in PBS. To definitively demonstrate that the lack of a direct dose–effect was not due to solubility issues of our lipophilic derivatives under the measured conditions, we prepared, as described, 1.4, 14, and 28 mN solutions of the compounds tested, i.e., from 14 to 280 times more concentrated than the upper limit (100 μN) concentration typically tested. The increase of the concentration was verified as proportional absorbance detected by UV-Vis spectra at 292 nm, as already reported [45] in Figure 4B. Hence, all tocopherols used were perfectly soluble in our experimental conditions, and the lack of a direct dose–effect relationship was not caused by the low solubility of tocopherols but, reasonably, was due to the complexity of the biological system, and mechanisms involved in the formation of ROS in the system under investigation.

### 3.3. Effect of Natural **α-Toc** and **δ-Toc**, Semi-Synthetic, **(δ-Toc)_2_S** and **(δ-Toc)_2_S_2_**, and NAC on TNFα-Induced ICAM-1 and Cl-2 Expression in HT29 Cells

**Figure 5:**
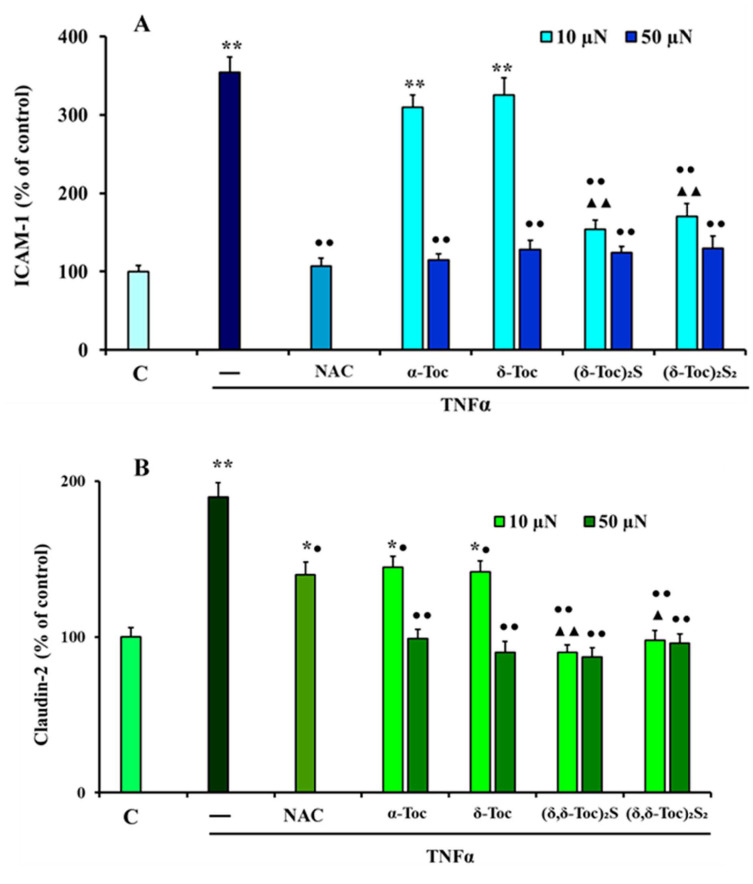
ICAM-1 and Cl-2 expression in HT29 cells pre-treated or not with natural, **α-Toc**, **δ-Toc**, semi-synthetic, **(δ-Toc)_2_S**, **(δ-Toc)_2_S_2_**, or NAC, and stimulated with TNFα. HT29, pre-treated or not with various concentrations (10–50 µN) of **α-Toc**, **δ-Toc**, **(δ-Toc)_2_S**, **(δ-Toc)_2_S_2_**, or 20 mM NAC, as reported in Materials and Methods, were stimulated or not for 24 h with 10 ng/mL TNFα. ICAM-1 levels (**A**) and Cl-2 levels (**B**) were assayed by ELISA kits in cell lysates. The values, expressed as the percentage of untreated and unstimulated cells (control, C), are the mean ±SEM of three experiments repeated in triplicate. * *p* ≤ 0.05, ** *p* ≤ 0.001 compared to C cells; ^●^ *p* ≤ 0.05, ^●●^ *p* ≤ 0.001 compared to untreated and TNFα-stimulated cells; ^▲^ *p* ≤ 0.05, ^▲▲^ *p* ≤ 0.001 compared to **α-Toc** and **δ-Toc** pre-treated, and TNFα-stimulated cells.

ICAM-1 and Cl-2 expression was detected in TNFα-stimulated HT29 cells pre-treated or not with intermediate concentrations of all tocopherol derivatives (10 and 50 µN) or 20 mM NAC. Figure 5A shows that TNFα up-regulated ICAM-1 expression by about 250%, compared to control cells. In addition, 10 µN **α-Toc**, **δ-Toc** were not able to prevent this, whereas, their highest concentration, both concentrations of **(δ-Toc)_2_S** and **(δ-Toc)_2_S_2_** and NAC, totally prevented the increase of ICAM-1 due to TNFα stimulation. This effect is in accordance with what was observed in the ROS production experiment, indicating a clear relationship between ICAM-1 expression and the intracellular redox state. TNFα also induced a significant increase, of about 95%, in Cl-2 expression, which was reduced, partially but significantly, by pre-treatment with 10 µN **α-Toc** and **δ-Toc** and NAC, compared to TNFα-stimulated cells, (Figure 4B). However, in these experimental conditions, Cl-2 levels were significantly higher than those detected in control cells, and not closely related to ROS levels, differently to those observed in ICAM-1 expression. The pre-treatment with 50 µN **α-Toc** and **δ-Toc,** and the *bis*-δ-tocopheryl sulfide and disulfide, **(δ-Toc)_2_S**, **(δ-Toc)_2_S_2_**, at both concentrations, was able to completely prevent the up-regulation of TNFα-induced Cl-2 expression.

## 4. Discussion

For the first time, it has been demonstrated that the pre-treatment of TNFα-stimulated cells with natural, **α-Toc** and **δ-Toc**, and semi-synthetic, **(δ-Toc)_2_S** and **(δ-Toc)_2_S_2_**, prevents the increase of ROS, Cl-2, and ICAM-1 levels.

This result deserves to be commented on as being deceptively obvious. The antioxidant activity of tocopherol is well known, and α-Toc is typically indicated, at least in vitro, as the more potent natural lipophilic natural antioxidant [46,47]. This is completely true when considering the chain breaking antioxidant activity, and the ability of tocopherols to react with peroxyl radicals (ROO*). In this reaction the kinetic inhibition constant of **α-Toc** is *k_inh_* = 3.2 × 10^6^ mol^−1^ s^−1^, the highest value measured for natural phenolic antioxidants. However, **δ-Toc**, due to the lack of two methyl groups on the aromatic ring, is roughly one order of magnitude less active than **α-Toc** as a chain breaking antioxidant with a *k_inh_* = 4.4 × 10^5^ mol^−1^ s^−1^ difference that is not observed in the measures reported in this manuscript. In the last two decades we have studied the effect of sulfur introduction on the activity of natural phenolic antioxidants [48,49,50,51,52,53,54,55,56,57,58,59,60]. In particular, the synthesis of **(δ-Toc)_2_S** and **(δ-Toc)_2_S_2_**, and related tocopherol sulfides and disulfides [34], has allowed us to measure their chain breaking antioxidant activity. Thus, we demonstrated that sulfides show an activity slightly worse than the corresponding tocopherol, for example for **(δ-Toc)_2_S** *k_inh_* = 2.2 × 10^5^ mol^−1^ s^−1^, but that a single tocopherol unit is operative as a peroxyl radical (ROO*) quencher. On the other hand, tocopheryl disulfides were a further order of magnitude less active than the corresponding tocopherols, **(δ-Toc)_2_S_2_** *k_inh_* = 1.5 × 10^4^ mol^−1^ s^−1^ [34]. Thus, it appears that the clear antioxidant effects observed and reported in this manuscript were not related to the ability of tocopherols for quenching ROO* radicals or other related oxygen centered radicals. This was confirmed by the results achieved with *bis*-δ-tocopheryl sulfide and disulfide, **(δ-Toc)_2_S, (δ-Toc)_2_S_2_**, which, very interestingly, exerted their activity with both the tocopheryl units.

The present study also shows that TNFα-induced oxidative stress can be involved in the up-regulation of Cl-2 and ICAM-1 expression in intestinal HT29 cells. These are cancer cells with characteristics of mature intestinal cells, and for this they represent a useful in vitro model for studying the antioxidant or barrier protective properties of food compounds [61,62]. Literature data show that in HT29 cells TNFα induces ROS production and Cl-2 and ICAM-1 expression up-regulation [17,18]. However, to our knowledge, there are no data regarding the role of natural and semi-synthetic vitamin E isoforms in the prevention of TNFα-induced oxidative stress and the expression of molecules involved in the adhesion processes and intestinal barrier function in these cells. Moreover, the literature lacks clear information on the relationship between oxidative stress and ICAM-1 and Cl-2 expression up-regulation in TNFα-stimulated HT29 cells. ICAM-1, a glycoprotein expressed on the surface of various cell types, is involved in leukocyte trans-migration to inflammatory sites, and it is up-regulated in the colonic epithelial cells of patients with IBD [63]. Instead, Cl-2, a member of the transmembrane tight-junctional proteins, forms cation selective channels, and is highly expressed in IBD, contributing to alteration of intestinal barrier function [20,64,65,66]. In TNFα-stimulated HT29 cells the enhancement of ROS levels seems to be involved in the up-regulation of ICAM-1 and Cl-2 expression, considering that NAC pre-treatment is able to down-regulate the increase of ROS, ICAM-1, and Cl-2 levels. Moreover, natural and semi-synthetic tocopheryl derivatives reduce TNFα-induced ROS production, and Cl-2 and ICAM-1 expression. However, the expression of ICAM-1 is directly related to intracellular redox state. In fact, the low concentrations of **α-Toc** and **δ-Toc** were unable to prevent both ROS and ICAM-1 increase in TNFα-stimulated cells; differently to what occurs when they are used at high concentrations. It should be noted that **(δ-Toc)_2_S** and **(δ-Toc)_2_S_2_** are more efficient than **α-Toc** and **δ-Toc** in preventing both ROS production and ICAM-1 expression increase. In fact, *bis*-δ-tocopheryl sulfide and disulfide, contrarily to **α-Toc** and **δ-Toc**, at both low and high concentrations, are able to completely prevent the increase of ROS levels and ICAM-1 expression increase, similarly to large excesses of NAC. These data indicate that in HT29 cells TNFα up-regulates the expression of ICAM-1 only by redox regulated mechanisms, differently to those verified in intestinal myofibroblasts, in which TNFα up-regulates ICAM-1 expression by both redox-dependent and-independent mechanisms [67,68]. On the other hand, both these mechanisms appear to be involved in the TNFα-induced up-regulation of Cl-2 expression that in HT29 cells is not directly related to ROS levels. In fact, pre-treatment with NAC or 10 µN **α-Toc or δ-Toc** partially prevents Cl-2 expression increase in TNFα-stimulated cells, although only NAC was able to suppress ROS increase. We can speculate that, in our experimental conditions, NAC mainly inhibits the redox-regulate mechanisms, by preventing ROS production, whereas 10 µN **α-Toc** or **δ-Toc** suppress the non-redox-regulate mechanisms by processes not directly related to the antioxidant action. Instead, **α-Toc** and **δ-Toc**, at the highest concentration, and **(δ-Toc)_2_S** and **(δ-Toc)_2_S_2_**, at both concentrations, are able to avoid the increase of ROS in a similar manner to NAC but, differently from this, are also able to prevent the total up-regulation of Cl-2 expression, probably through the inhibition of TNFα-related redox- and non-redox-dependent mechanisms. **(δ-Toc)_2_S** and **(δ-Toc)_2_S_2_** are much more effective than natural vitamin E isoforms in the prevention of Cl-2 up-regulation in TNFα-stimulated HT29 cells, similarly to that which occurs for ROS production and ICAM-1 expression. Altogether, these results suggest that, in TNFα-stimulated HT29 cells, natural tocopherols and semi-synthetic tocopheryl sulfide and disulfide prevent the increase of expression of ICAM-1 and Cl-2 thanks to their antioxidant properties. However, vitamin E can also suppress Cl-2 expression by its ability to regulate redox-independent mechanisms. It is possible that the pre-treatment with the various tocopheryl derivatives in TNFα-stimulated HT29 cells prevents the oxidative stress, and then the activation of redox-sensitive regulatory transcription factors, such as NF-kB. In fact, TNFα administration to an animal model can up-regulate ICAM-1 and Cl-2 expression by NF-kB activation [69,70], as in various types of cells stimulated with TNFα [71,72,73]. Vitamin E reduces the phenobarbital-induced NF-kB activation in rats [74], and **δ-Toc** inhibits the phosphorylation of a kinase essential for TNFα-induced NF-kB activation in macrophages [75]. Moreover, **δ-Toc** prevents constitutive NF-kB activation in pancreatic cancer [76], and **α-Toc** succinate reduces NF-kB activity and ICAM-1 expression in prostate cancer cells [77]. Vitamin E can also modulate biological responses and signaling pathways, by mechanisms independent of its antioxidant role [1,78]. Indeed, vitamin E, by inhibiting protein kinase C (PKC) activity, down-regulates in cells many events, including inflammation, proliferation, adhesion, and migration [79]. In HT29/B6 cells, a sub-clone of the HT29 cell line, TNFα mediates the up-regulation of Cl-2 expression by activating the phosphatidylinositol-3-kinase (PI3K) pathway and NF-kB [80,81]. We also speculate that, in our experimental conditions TNFα could up-regulate Cl-2 expression by mechanisms that involved NF-kB and PI3K activation, and that tocopherols, by their non-antioxidant properties, could affect Cl-2 expression by inhibiting PKC that may be activated downstream of PI3K [82,83]. Vitamin E down-regulates oxidative stress and adhesion molecule expression in the inflammatory processes present in various diseases [84,85,86,87], and reduces ICAM-1 in cultured microglial cells [88], as well as in human aortic endothelial cells [89]. Moreover, vitamin E ameliorates oxidative stress in the intestine of animals, and maintains the integrity and the correct functionality of the intestinal barrier [90,91]. The administration of certain vitamin E derivatives to animals with experimental colitis decreases colonic injury, intestinal neutrophil infiltration, and TNFα levels, and regulates levels of interleukins [29,30]. In IBD patients, the increase of ICAM-1 is involved in the massive recruitment of leukocytes to intestinal inflammatory sites, while the enhancement of Cl-2 can induce the decrease and/or redistribution of epithelial tight junction proteins, leading to cytokine level increases and barrier dysfunction [16,92,93,94]. For this, TNFα, by up-regulating ICAM-1 and Cl-2, contributes to exacerbating the inflammatory state and altering the epithelial barrier function. The natural tocopherols and semi-synthetic tocopheryl sulfur containing derivatives used in this study can mitigate the effect of TNFα, a cytokine with a key role in the chronic intestinal inflammation that characterizes IBD, by preventing the oxidative stress and the up-regulation of ICAM-1 and Cl-2.

## 5. Conclusions

**Figure 6:**
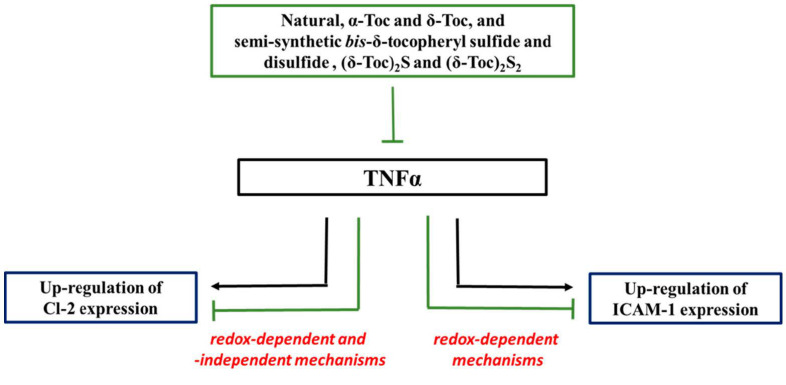
Schematic diagram illustrating the role of natural, **α-Toc**, **δ-Toc**, and semi-synthetic **(δ-Toc)_2_S** and **(δ-Toc)_2_S_2_** on TNFα-induced ICAM-1 and Cl-2 expression in HT29 cells. TNFα up-regulates ICAM-1 expression by redox-regulated mechanisms, and Cl-2 expression by redox- and non-redox-regulated mechanisms. Natural tocopherols and semi-synthetic δ-tocopheryl sulfide and disulfide inhibit both these mechanisms and prevent the up-regulation of ICAM-1 and Cl-2 expression.

This study shows, for the first time, that natural tocopherols, **α-Toc** and **δ-Toc**, and, even more efficiently, semi-synthetic δ-tocopheryl sulfide and disulfide, **(δ-Toc)_2_S** and **(δ-Toc)_2_S_2_**, prevent oxidative stress and ICAM-1 and Cl-2 expression up-regulation, in TNFα-stimulated HT29 cells, by their antioxidant properties. This is particularly evident for ICAM-1, because of the presence of a strong relationship between ICAM-1 expression and intracellular redox state. Data obtained clearly indicate that the effect of **α-Toc** and **δ-Toc**, and **(δ-Toc)_2_S** and **(δ-Toc)_2_S_2_**, is not related to their well-known peroxyl radical scavenger ability. Conversely, TNFα-induced Cl-2 expression is due to redox- and non-redox-dependent mechanisms, and the two tocopherols, as well as the semi-synthetic δ-tocopheryl sulfide and disulfide tested, are able to inhibit both mechanisms involved. In any of the registered effects, **(δ-Toc)_2_S** and **(δ-Toc)_2_S_2_** demonstrated that both their tocopheryl units participate in the biological effect, having an activity similar or better than that of **α-Toc** and **δ-Toc** used in doubled molar concentration (Figure 6).

We hope that in due course the additional investigations in our laboratories can confirm the beneficial role of **(δ-Toc)_2_S** and **(δ-Toc)_2_S_2_** in the intestinal inflammatory diseases, in which are present high levels of TNFα.

## Data Availability

Not Applicable.
